# Effects of Acute Tryptophan Depletion on Prefrontal-Amygdala Connectivity While Viewing Facial Signals of Aggression

**DOI:** 10.1016/j.biopsych.2011.07.033

**Published:** 2012-01-01

**Authors:** Luca Passamonti, Molly J. Crockett, Annemieke M. Apergis-Schoute, Luke Clark, James B. Rowe, Andrew J. Calder, Trevor W. Robbins

**Affiliations:** a Unità di Ricerca Neuroimmagini, Consiglio Nazionale delle Ricerche, Catanzaro, Italy; b Cognition and Brain Sciences Unit, Medical Research Council, University of Cambridge, Cambridge, United Kingdom; c Behavioural and Clinical Neuroscience Institute, University of Cambridge, Cambridge, United Kingdom; d Departments of Experimental Psychology, University of Cambridge, Cambridge, United Kingdom; e Department of Clinical Neurosciences, University of Cambridge, Cambridge, United Kingdom

**Keywords:** 5-HT, amygdala, anterior cingulate cortex, effective connectivity, fMRI, violence

## Abstract

**Background:**

Reduced levels of serotonin (5-HT) within prefrontal cortex (PFC)–amygdala circuits have long been implicated in impulsive aggression. However, whether lowering 5-HT alters the dynamic interplay between the PFC and the amygdala has not been directly tested in humans. It is known that manipulating 5-HT via acute tryptophan depletion (ATD) causes variable effects on brain responses to a variety of emotional stimuli, but it remains unclear whether ATD affects functional connectivity in neural networks involved in processing social signals of aggression (e.g., angry faces).

**Methods:**

Thirty healthy individuals were enrolled in a randomized, double-blind, placebo-controlled ATD study. On each treatment, brain responses to angry, sad, and neutral faces were measured with functional magnetic resonance imaging. Two methods (psycho-physiological-interaction in a general linear model and dynamic causal modeling) were used to assess the impact of ATD on the functional connectivity between PFC and amygdala.

**Results:**

Data from 19 subjects were available for the final analyses. A whole-brain psycho-physiological-interaction in a general linear model showed that ATD significantly modulated the connectivity between the amygdala and two PFC regions (ventral anterior cingulate cortex and ventrolateral PFC) when processing angry vs. neutral and angry vs. sad but not sad vs. neutral faces. Dynamic causal modeling corroborated and extended these findings by showing that 5-HT depletion reduced the influence of processing angry vs. neutral faces on circuits within PFC and on PFC–amygdala pathways.

**Conclusions:**

We provide strong support for neurobiological accounts positing that 5-HT significantly influences PFC**–**amygdala circuits implicated in aggression and other affective behaviors.

Serotoninergic abnormalities within prefrontal cortex (PFC)–amygdala circuits are thought to underlie several psychiatric disorders characterized by emotional dysregulation, including aggression (1,2). Two lines of research support this view.

First, low levels of serotonin (5-HT) metabolites in the cerebrospinal fluid and blunted responses to serotoninergic drugs have been reported in aggressive individuals (3–7). Furthermore, 5-HT manipulations (dietary and pharmacologic) alter aggressive behavior in animals, healthy volunteers, and individuals with history of aggression (8–12). Aggression is also associated with genetic polymorphisms affecting the 5-HT system, including the tryptophan hydroxylase and monoamine oxidase-A (MAO-A) (13,14). These studies have pinpointed a specific role for 5-HT in impulsive-reactive aggression (as distinct from instrumental aggression) (7,15,16), suggesting that 5-HT is fundamental for regulating emotional behavior.

Second, research investigating the neural basis of aggression has implicated abnormalities within PFC–amygdala circuits. These studies have frequently used angry faces as stimuli because they are universal signals of threat and tend to evoke hostile feelings in the beholder, particularly when interpreted as a provocation (17); nonetheless, it is worth noting that experiencing angry feelings is different from processing angry faces. Heightened amygdala reactivity to angry faces or aggressive acts has been observed in patients with violent outbursts, including intermittent explosive disorder (IED) and borderline personality disorder (BPD) (18–21), as well as in violent offenders (22). Evidence for PFC involvement comes from patients with lesions of the orbitofrontal cortex (OFC) who often display heightened levels of aggression (23) and from work showing reduced medial PFC in response to anger induction in BPD patients and depressed patients with anger attacks (19,24). However, some studies have failed to detect hypoactive PFC responses in aggressive individuals (22), suggesting that PFC abnormalities in aggression may be more complex than simple hypoactivity (25); for instance, connectivity between PFC and subcortical regions may be impaired. There is evidence suggesting that functional interactions between the PFC and amygdala are indeed critical for processing facial signals of anger.

One study reported reduced functional coupling between amygdala and OFC when viewing angry faces in patients with IED and BPD relative to healthy control subjects (18,26). Another study in healthy volunteers showed that reward drive, a personality trait linked to aggression (27–30), predicted reduced connectivity between the ventral anterior cingulate cortex (vACC) and amygdala when processing angry vs. neutral faces (31). Although it has been suggested that 5-HT plays a role in facilitating functional interactions between PFC and amygdala (1,2), direct evidence for this hypothesis is limited. Comparative research has shown reduced levels of 5-HT within the PFC of aggressive animals (32–35), and a positron emission tomography study reported reduced metabolism in OFC and ACC in response to a 5-HT challenge in impulsive aggressive patients (36). However, no study has examined the effects of manipulating 5-HT on functional connectivity within the neural networks linked to aggressive behavior yet.

To investigate 5-HT function in humans, researchers frequently use a dietary procedure called acute tryptophan depletion (ATD) (37–39). Tryptophan is the chemical precursor of 5-HT and is only obtained through the diet; hence, ingesting an alimentary mixture without tryptophan rapidly decreases plasma and brain levels of 5-HT (40–43). In the current study, we used functional magnetic resonance imaging (fMRI) to explore how ATD influences brain circuitry involved in processing facial expressions of anger. Because reward drive strongly influences regions underlying anger processing (31,44–46), we also investigated whether this personality measure interacted with the effects of ATD on brain function. Although previous research has examined the effects of ATD on neural responses to emotional facial expressions (39,47–50) and has shown that OFC activity during resting state is reduced by 5-HT depletion (51), the results have been variable; furthermore, no study has specifically examined the neural responses to angry faces, an ecologically valid stimulus for probing brain networks involved in reactive aggression (52).

We predicted that ATD would affect the connectivity among the amygdala, OFC, vACC, and/or ventrolateral PFC (VLPFC), a set of tightly interconnected regions rich in 5-HT and involved in controlling aggression and, more generally, in emotional regulation (1–2,53–60). In particular, we hypothesized that the negative change in connectivity previously detected between the amygdala and vACC (31), when viewing angry vs. neutral faces, would be altered (reduced or reverted) by ATD. Formal analyses of the effective connectivity allow heightened understanding of how ATD modulates the circuitry underlying processing angry faces and of how brain effects driven by 5-HT manipulation may interact with personality measures (e.g., reward drive) linked to aggression.

To achieve these objectives, two different but complementary methods for investigating brain effective connectivity were used: psycho-physiological-interactions (PPI) in a general linear model (GLM) and dynamic causal modeling (DCM). PPI-GLM is an anatomically unconstrained (whole-brain), data-driven approach but does not provide the directionality of any changes in connectivity between regions (61). DCM is an alternative method for analyzing PPI within hypothesis-driven models that overcomes this limitation (62). Specifically, DCM explains the activity of groups of regions in terms of 1) “driving” inputs (here, processing a face, regardless of the expression) directly triggering the response in one or more areas of the network; 2) a psychological context (here, viewing angry vs. neutral faces) acting on “intrinsic” (anatomic) pathways and changing the pattern of functional connectivity between regions (62).

## Methods and Materials

### Participants

Thirty healthy volunteers (17 women; mean age: 25.1 ± 3.2 years) gave their written informed consent and were financially compensated for participating in this study that was approved by the Cambridgeshire Research Ethics Committee. Exclusion criteria included history of cardiac, hepatic, renal, pulmonary, gastrointestinal, and neurological disorders; medication use; and personal or family history of major depression, bipolar disorder, or other psychiatric illness. After a screening interview, participants were assigned to receive either the tryptophan-depleting drink or the placebo mixture on the first session in a double-blind, counterbalanced order. Participants attended two distinct sessions, separated by at least 1 week. At each session, participants completed a self-report measure of the mood state (see Supplement 1) and the fMRI task (described subsequently). On the first session, participants completed a personality questionnaire assessing the reward drive (27).

### Serotonin Dietary Manipulation

We used ATD to temporarily lower plasma and brain 5-HT levels in a randomized, double-blind, placebo-controlled, within-subjects, counterbalanced design, as previously described (63) (Supplement 1). Blood samples were collected at baseline and before scanning to confirm the plasma tryptophan depletion.

### fMRI Task

Participants categorized the sex of angry, sad, and neutral faces (50% female, 30 identities). Faces were selected from two stimulus sets (64) (http://www.macbrain.org) on the basis of emotional ratings from an independent sample (65). Emotional ratings were also obtained from all participants in the study during each treatment (ATD and placebo). Stimuli were grouped in 17.5-sec epochs containing five faces from the same category (angry, sad, neutral) intermixed with 5 null events (fixation cross). Each face trial comprised a 1000-msec presentation of a face followed by a fixation cross (750 msec). Null events constituted a 1750-msec presentation of the same fixation cross. Stimuli within each epoch were pseudo-randomized with respect to trial type (face or null events), face sex, and identity; no more than three consecutive trials were of the same trial type. Pseudo-randomization enhances design efficiency while preserving the unpredictability of stimulus onsets in naïve participants. There were 12 epochs for each category (60 angry, 60 sad, 60 neutral faces; total task duration: 10 min, 30 sec) and 2 orders of presentation, counterbalanced across treatments and subjects. Reaction times (RT) and accuracy for gender discriminations were recorded throughout the task.

### Image Acquisition and Preprocessing

The fMRI was performed on a 3-Tesla Unit (Tim Trio; Siemens, Surrey, England) set at the Wolfson Brain Imaging Centre. Whole-brain data were acquired with echo-planar imaging (EPI) sensitive to the blood oxygenation level–dependent signal contrast (32 axial slices, 3-mm thickness; repetition time, 2000 msec; echo time, 30 msec; voxel size, 3 × 3 × 3 mm; field of view, 192 mm). Data were analyzed with SPM8 (http://www.fil.ion.ucl.ac.uk/spm). EPIs were sinc-interpolated in time to adjust for slice time differences and realigned to the first scan by rigid body transformations to correct for head movements. The mean EPI was computed for each subject and visually inspected to check for excessive signal dropout in the medial temporal cortices and OFC. EPIs were normalized to the T1 standard template in the Montreal Neurological Institute (MNI) space using linear and nonlinear transformations and were smoothed with a Gaussian kernel of full width at half maximum of 8 mm.

### Analyses of Regional Effects

For each participant, a GLM assessed regionally specific effects of task parameters on blood oxygenation level–dependent indices of activation. The first-level model included four experimental factors (angry, sad, neutral faces, and null events) and six realignment parameters as effects of no interest to remove residual motion-related variance. Low-frequency signal drift was eliminated using a high-pass filter (cutoff, 128-sec) and an autoregressive model [AR(1)] was applied to correct for voxels' autocorrelations. Subjects' specific contrast images were generated for the angry > neutral comparison and entered into a second-level GLM investigating the main effect of treatment (ATD > placebo, placebo > ATD; paired *t* tests); similar analyses addressed the main effect of treatment for sad > neutral and angry > sad faces comparisons. These random-effects analyses assessed effects on the basis of intraparticipant variance and allowed inferences about the population that the participants were drawn from (66).

Two approaches for thresholding second-level maps were applied. First, for a priori regions of interest (ROIs), the threshold was set at *p* < .05, family-wise error (FWE) correction for multiple comparisons in small volumes (i.e., small volume correction [svc]) (67,68). The amygdala, ACC, insula, VLPFC, and OFC were defined as ROIs given their fundamental role in aggression and emotional behavior in general (1,2). All ROIs were defined using the “aal.02” atlas for automated anatomic labeling (69). Second, we reported regions that were not predicted a priori but met a threshold of *p* < .001, uncorrected, greater than 10 contiguous voxels.

### Effective Connectivity Analyses

#### Psychophysiological Interaction in a GLM

The physiologic connectivity between two regions can vary with the psychological context (61). In this study, the connectivity arising from the presentation of faces could vary depending on whether the context is angry vs. neutral, for example. This constitutes a Psychophysiological Interaction (PPI) (61). We sought to identify “target” areas that had a differential connectivity with a “source” (amygdala) as a function of each context and treatment (ATD vs. placebo).

Separate PPIs were carried out for angry vs. neutral, sad vs. neutral, or angry vs. sad contexts, using either the right or left amygdala seed. All PPIs used the same procedure, so it is described for the right amygdala seed and the angry vs. neutral contexts alone. Data from the amygdala were extracted from a 8-mm sphere, constructed around the MNI center of mass derived from the anatomical definition of the amygdala according to the aal.02 atlas (right amygdala, x 28, y 0, z –20; left amygdala, x –24, y –2, z –18) (69). The time series for each participant was computed using the first eigenvariate from all voxels' time series within the sphere and then deconvolved to estimate a “neuronal time series” (70). The PPI regressor was calculated as the element-by-element product of the right amygdala neuronal time series and a vector coding for the main effect of task (1 for angry, –1 for neutral, 0 for sad faces, 0 for null events). This product was reconvolved by the canonical hemodynamic response function (HRF). The first-level model included the main effect of the task convolved by the HRF and six movement parameters as effects of no interest. Subject-specific PPI contrast images were computed and entered into a second-level GLM that identified those brain areas for which the change in connectivity with the amygdala (for the angry vs. neutral contrast) was modulated by treatment (ATD vs. placebo; paired *t* test). We also examined whether participants' sex, individual differences in reward drive or plasma tryptophan levels modulated any ATD effect on brain connectivity. The same statistical approaches previously described for the analyses of regional effects were employed for second-level connectivity maps.

#### DCM

Distinct DCM analyses were conducted for each treatment (ATD, placebo) using the following procedure. Models were created on the basis of the neural circuit identified by PPI-GLM (see PPI in Results). Data from the right amygdala, used in PPI-GLM, were also employed in DCM. For the right vACC and VLFPC, first eigenvariates were extracted from 15-mm spheres centered on the local maxima identified by PPI. The standard model included “intrinsic” bidirectional connections among the amygdala, vACC, and VLFPC, according with the anatomy of this circuit (55–60). “Intrinsic” connections (DCM matrix “A”) represent the fixed coupling between regions in absence of any experimental manipulation. Responses in a network can be changed in two ways. First, “driving inputs” (all faces vs. fixation, DCM matrix “C”) can directly influence individual or groups of regions within the network. Second, changes in the psychological context (anger vs. neutral faces; DCM matrix “B”) may modulate the “intrinsic” connections.

We specified 49 biologically plausible models in which the number and location of “driving inputs” and psychological modulators systematically varied. As recently described (71), models were grouped in “Meta-Families” (A,B,C) based on where “driving inputs” entered the network. These entered the circuit via 3, 2, or 1 region(s) (Meta-Families, A, B, or C, respectively). Each Meta-Family comprised three families. This further grouping reflected differences in the number and location of connections at which angry vs. neutral modulated bidirectional connections across either 3, 2, or 1 pathway(s). Figures S1–S3 in Supplement 1 display all models.

Random-effects Bayesian model selection (RFX-BMS, SPM8/DCM10 toolbox) (72–74) under ATD and placebo identified the most likely model. However, the relative model evidences of models can be only compared within the same data sets (ATD and placebo, separately) and not across treatments. This does not prevent DCM from being used to test pharmacologic effects on brain networks, especially where relative model differences are reversed between groups or drug conditions, as previously demonstrated (75). RFX-BMS reports the expected posterior probability (how likely it is that a specific model generated the data of a randomly chosen subject) and the exceedance probability (how much each model is more likely than any other model). These two values from RFX-BMS are in themselves statistical inferences (statements of relative probabilities) and not absolute parameters of the goodness of model fit for a data set. RFX-BMS does not assume that the optimal model is the most likely for all subjects individually and is therefore less susceptible to outliers than fixed-effects methods (72–74). This implies that model selection is relativistic, assessing models against each other. Furthermore, the expected and exceedance probabilities of an individual model will be reduced as the number of models increase. Hence, we only examined a set of highly plausible models and grouped those with shared features into Families and Meta-Families.

## Results

### Participants

Eleven subjects (8 women) were excluded from final analyses for at least one of the following reasons: 1) head movements greater than 2 mm during one or both fMRI sessions; 2) excessive signal dropout in the medial temporal regions and/or OFC, as assessed by a careful visual inspection of the mean EPI. The final group thus included 19 subjects (9 women; mean age: 24.5 ± SD 3.3 years).

### Serotonin Manipulation

ATD significantly reduced both plasma tryptophan levels and the ratio between tryptophan and other large neutral amino acids (TRP:ΣLNAA). A repeated-measures analysis of variance revealed a significant 2-way interaction between treatment (ATD, placebo) and time point (baseline, prescan [+5.5 h since baseline]), resulting from significant reductions in total tryptophan levels [*F*(1,18) = 108.5, *p <* .0001) and TRP:ΣLNAA ratio [*F*(1,18) = 28.6, *p <* .0001] 5.5 h following ATD relative to placebo (Figure S4 in Supplement 1).

### fMRI Regional Effects

Paired *t* tests exploring the main effect of treatment (ATD > placebo, placebo > ATD) for each contrast (angry > neutral, sad > neutral, angry > sad) did not identify significant activations in any area including our a priori ROIs. For regional effects during placebo and ATD separately, see Supplement 1.

### Effective Connectivity 1: PPI in the GLM

**Figure 1:**
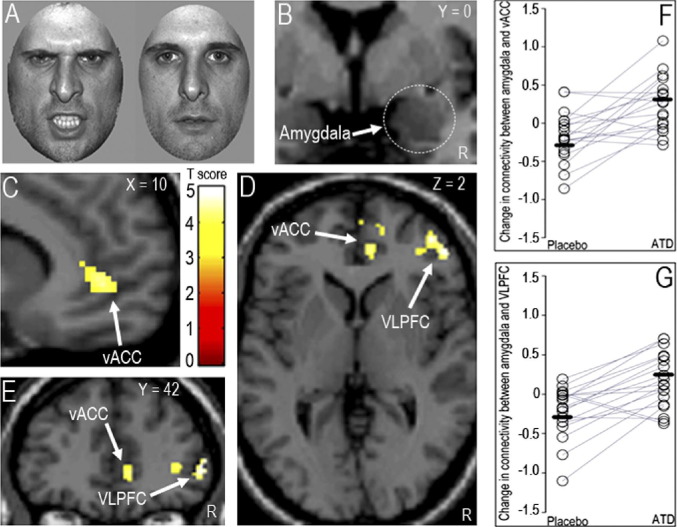
**(A)** Examples of angry and neutral faces during the task. **(B)** “Source” for Psycho-Physiological Interactions (PPI) (8-mm right amygdala sphere). **(C–E)** PPI Statistical Parametrical Maps (SPM). These SPM maps demonstrate that the ventral anterior cingulate cortex (vACC) and the ventrolateral prefrontal cortex (VLPFC) are the only two regions in the whole brain that are connected with the right amygdala as function of viewing angry vs. neutral faces and treatment (acute tryptophan depletion [ATD] and placebo; paired *t* tests). Slices shown (x, y, z) are in the Montreal Neurological Institute space. For display purposes, threshold is set at *p <* .001, uncorrected; the effects are significant at *p <* .05, family-wise error, small volume correction. The color bar represents *t* statistics. R, right hemisphere. **(F, G)** Plots of the individual data for the local maxima displayed in panels C and D, respectively. Significant differences between placebo and ATD reflect a negative change in the connectivity between the amygdala and vACC/VLPFC during placebo and a positive change in connectivity between the same regions during ATD. Black lines represent mean values for each treatment (placebo, ATD).

**Figure 2:**
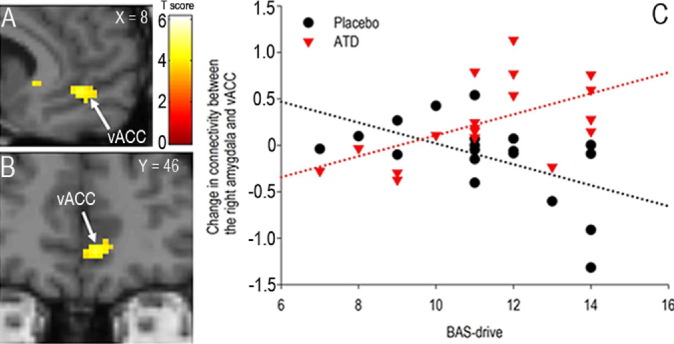
**(A, B)** The ventral anterior cingulate cortex (vACC) is connected with the right amygdala as a function of treatment (acute tryptophan depletion [ATD], placebo) and reward drive (BAS, behavioral activation system; treatment by personality interaction; for angry vs. neutral faces). Slices (x, y) are in the Montreal Neurological Institute space. For display purposes, threshold is set at *p <* .001, uncorrected; the effects are significant at *p <* .05, family-wise error, small volume correction. The color bar represents *t* statistics. **(C)** Plot of individual data for the local maxima displayed in panels **A** and **B**. High-BAS-drive individuals displayed the largest effect of ATD on amygdala-vACC connectivity.

Despite no effect on local brain activity, self-reported mood, fMRI task performance or ratings of facial expressions (Supplement 1), the PPI for the right amygdala seed and for angry vs. neutral faces and ATD vs. placebo comparison identified the right vACC and VLPFC (MNI local maxima, vACC: x 10, y 44, z 2; T = 4.1; *p <* .04, FWE, svc; VLPFC: x 52, y 42, z 2; T = 5.0; *p <* .01, FWE, svc, Figure 1C-E). Of note, these were the only two regions in the whole brain showing a change in connectivity with the right amygdala as a function of viewing angry faces and ATD. A similar effect was found when using the left amygdala seed, although at a lower threshold (see Supplement 1). Importantly, ATD significantly reverted the negative change in PFC–amygdala connectivity identified under placebo (for angry vs. neutral faces; Figure 1F–1G). Furthermore, these results were driven by the angry face context, because the PPI for angry vs. sad produced similar results to those identified for angry vs. neutral and for both the right and left amygdala seed (see Supplement 1), whereas no significant effects were found for the sad vs. neutral PPI. The investigation of the interaction between treatment and reward drive demonstrated a significant PPI for the right amygdala seed and for angry vs. neutral in the vACC (x 8, y 46, z 4, T = 4.6, *p <* .05, FWE, svc; Figure 2A–2B). Individuals with high reward drive displayed the highest magnitude of the ATD effect relative to placebo (Figure 2C). Finally, no brain areas were identified for the opposite comparison (placebo vs. ATD) for each contrast (angry vs. neutral, angry vs. sad, sad vs. neutral). For connectivity effects under placebo and ATD separately, for the ATD by sex interaction and for the effect of plasma tryptophan levels, see Supplement 1.

### Analysis of Effective Connectivity 2: DCM

**Figure 3:**
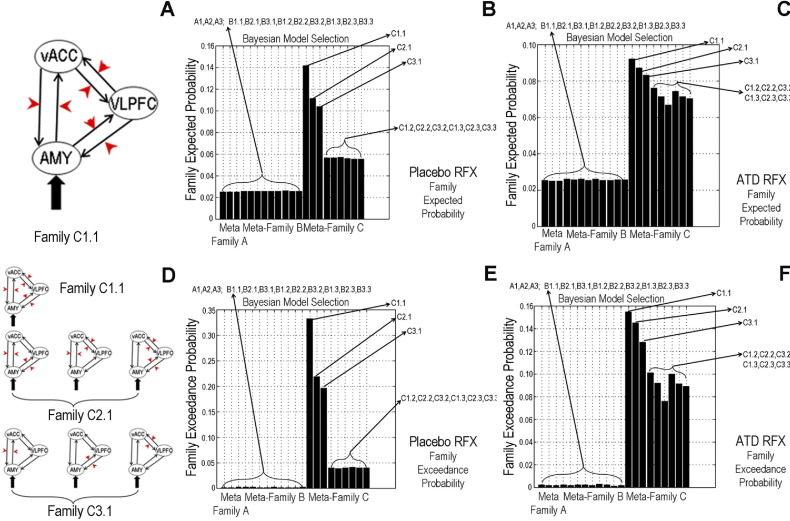
**(A)** Preferred model during placebo. The amygdala (AMY) is the only region where driving inputs (all faces vs. fixation, black “thick” arrows) start the “perturbation” of the network. In this model, the contextual modulator (angry vs. neutral faces, red arrows) influences all neural pathways linking the ventral anterior cingulate cortex (vACC), ventrolateral prefrontal cortex (VLPFC), and AMY. The intrinsic connectivity (black “thin” arrows) represents the couplings between regions irrespective of any experimental manipulation and are modeled as reciprocal connections between all three regions. **(B, C)** Expected probability and **(E and F)** exceedance probability for all 49 models shown in Supplement 1 Figures S1–S3 using random-effects (RFX) Bayesian model selection during placebo and acute tryptophan depletion (ATD). Compared with placebo, under ATD, model C1.1 has lower expected and exceedance probabilities; at the same time, model C2.1 and C3.1 **(D)** became more likely. These latter models are characterized by a smaller number of contextual modulators (affecting two or one couples of specific pathways) compared with model C1.1 where contextual modulators affect all possible pathways.

For placebo, RFX-BMS indicated evidence favoring model C1.1 (Figure 3B–E). In this family (Figure 3A), driving inputs (all faces) entered the system via the amygdala alone, whereas the angry vs. neutral modulator affected bidirectional connections in all three pathways. Hence, during placebo, the effect of the task is distributed within internal PFC circuitry and across PFC–amygdala connections.

Under ATD, the expected and exceedance probabilities of C1.1 were reduced (Figure 3C–F) with increased expected and exceedance probabilities of the two models (C2.1, C3.1) (Figure 3D) in which the contextual modulator acted on two or one bidirectional connections. Furthermore, during ATD, another family of six models (Figure S3 in Supplement 1) became more likely than under placebo, in which driving inputs “perturbed” the network via either the VLPFC or vACC alone.

To summarize, ATD reduced not only the number of PFC–amygdala pathways affected by processing angry faces but also the location where face information entered the network.

## Discussion

We demonstrated that ATD significantly altered the functional connectivity between the amygdala and right vACC and VLPFC when processing angry compared with neutral faces. Additional PPI analyses showed that the right amygdala–vACC connectivity was modulated by individual differences in reward drive, a personality trait linked to aggression (27–30). DCM supported and extended these findings. The best data-fitting model under placebo was characterized by diffuse modulation of angry faces on internal PFC pathways (vACC ↔ VLPFC) and on PFC-amygdala reciprocal connections (vACC ↔ amygdala, VLPFC ↔ amygdala). ATD made this model relatively less likely while enhancing the probability of alternative models in which angry faces acted on a reduced number of pathways.

The evidence that ATD altered connectivity within PFC–amygdala circuits when processing angry faces, particularly in high reward-drive people, supports neurobiological models positing that 5-HT facilitates the PFC in suppressing the negative emotions, generated in the amygdala, that are associated with aggression and other emotional behaviors (1–2,32,34,76). Although underlying molecular mechanisms are poorly understood, dysfunctions in 5-HT reuptake (5-HT transporter, 5-HTT), catabolism (MAO-A), and postsynaptic receptors (5-HT_2A_ and 5-HT_2C_) are thought to disrupt PFC top-down control over the amygdala, resulting in emotional dysregulation (1,77–83). This view is supported by neuroimaging research demonstrating that genetic polymorphisms of 5-HTT and MAO-A modulate circuits underlying emotions and impulsivity (79,81–87). The 5-HTT polymorphism is also known to interact with ATD in modulating brain metabolic abnormalities in the vACC and amygdala of patients with major depressive disorders (88). Furthermore, selective binding of 5-HTT or 5-HT_2A_/5-HT_2C_ receptor agonists is changed in the ACC and OFC of individuals with BPD or IED, two psychiatric conditions characterized by extreme violence (89,90). Overall, these results suggest possible mechanisms by which ATD may influence PFC–amygdala connectivity and, consequently, aggression and other affective behaviors. However, prudence is warranted when attributing specific molecular mechanisms to the ATD effect on functional connectivity because we did not measure 5-HT metabolism or receptor function. Because of the diffuse cortical–subcortical projections of the serotoninergic raphe-nuclei, it could also be that ATD affected PFC–amygdala connectivity via its effects on single regions; for example, acting on intra-amygdala inhibitory interneurons (91). However, the lack of ATD effects on isolated brain activities does not support this hypothesis. In contrast, negative regional findings highlight the importance of assessing functional connectivity when exploring brain effects of pharmacologic manipulations (92).

Another issue concerns the roles that vACC and VLPFC may play in modulating amygdala function. The vACC is a key region in regulating emotional behavior, given its abundant anatomic links with the amygdala and brainstem nuclei responsible for the visceral and endocrine responses associated with salient events (58–60). On the other hand, widespread connections of the VLPFC with both cognitive (e.g., dorsolateral PFC, inferior parietal lobule) and limbic regions (including vACC and amygdala) allow it to integrate executive functions with affective stimuli (93,94). This flow of communication between the amygdala, vACC and VLPFC may be one way in which emotions influence cognition and vice versa. ATD significantly affected these neural interactions, which might explain the profound impact of 5-HT manipulations on basic emotional reactions as well as on complex decision-making processes necessitating an high-level integration of affective and abstract informations (63,95–98).

It is also noteworthy that during placebo, facial informations (“driving inputs”) entered the circuit via the amygdala alone, while under ATD the role of the amygdala was relatively reduced, and other routes by which faces could activate the network became more likely (via either the VLPFC or vACC alone). Hence, ATD may also alter the more fundamental neural mechanisms by which faces engage the amygdala and PFC regions. There is evidence, from electrophysiologic studies in humans and cell recordings in monkeys, that neurons within the amygdala, vACC, and VLPFC show a similar fast response (∼110–220 msec) to faces, although it is unknown whether the rapid activation of PFC neurons occurs via the amygdala or is mediated via extra-amygdala pathways (99–102). RFX-BMS strongly supported the first hypothesis under placebo, but less so likely under ATD. However, in a prior nonpharmacologic DCM study, we found that facial informations entered both the amygdala and vACC, suggesting that the vACC response to faces may be triggered via extra-amygdala routes (31). Nonetheless, we did not previously include the VLPFC, and this, along with a larger model space selection, may have led to different model selection across experiments (72–74).

Questions may arise regarding the absence of effects for sad faces given that previous ATD fMRI studies have reported both significant (48,50) and nonsignificant findings for this stimulus (39). Because the former result may depend on a familial or personal history of depression (50), it is worth emphasizing that we excluded subjects with these histories. Nonetheless, the specificity of our findings for angry faces remains to be verified in future experiments comparing anger with other emotions as fear, disgust, surprise, or happiness.

Lastly, it might be argued that our results reflect a global ATD-effect on cerebral blood flow rather than specific mechanisms on brain 5-HT levels, as recently surmised (103). However, this possibility appears unlikely because we found functional connectivity changes that were strikingly restricted to particular PFC areas and critically depended on a specific task. A general vascular effect is unlikely to produce such regional- and task-distinct results. Furthermore, although we acknowledge that ATD also triggers additional molecular mechanisms that are not directly related to 5-HT (103), it is important to note that the overall current evidence is still favoring a predominant ATD effect on serotoninergic function (104).

In conclusion, this research represents a significant advance in building realistic circuitry models exploring brain effects of serotoninergic manipulation. These models may have clear implications for a broad range of psychiatric disorders, including aggression, that strongly depend on 5-HT dysfunctions.
