# Supplementary material for: Effects of Acute Tryptophan Depletion on Prefrontal-Amygdala Connectivity While Viewing Facial Signals of Aggression
Source: Biol Psychiatry. 2012 Jan 1;71(1):36–43. doi: 10.1016/j.biopsych.2011.07.033 (PMC3368260; doi:10.1016/j.biopsych.2011.07.033)
Supplement: Supplement 1 [file mmc1.pdf]

Effects of Acute Tryptophan Depletion on Prefrontal-Amygdala Connectivity While Viewing Facial Signals of Aggression

Supplemental Information

Supplemental Methods & Materials

Self-reported mood and serotonin manipulation procedure

Upon arrival, subjects completed the Positive and Negative Affect Scale (PANAS) questionnaire (1), gave a blood sample and ingested either placebo or tryptophan-depleting amino acid drink. Next, a resting period of approximately 5.5 hours was observed to achieve stable and low tryptophan levels (2); after that, PANAS and a second blood sample were re-collected and finally subjects participated in the functional magnetic resonance imaging (fMRI) session. In acute tryptophan depletion (ATD), tryptophan is depleted by ingestion of a liquid amino acid load that does not contain tryptophan but that includes other large neutral amino acids. Participants were asked to abstain from food, alcohol and caffeine from 00:00 before each session which commenced between 08:30 and 10:30 am. Amino acid mixtures (prepared by SHS International, Liverpool, UK; (3)) were as follows: Tryptophan depletion: L-alanine, 4.1 g; L-arginine, 3.7 g; L-cystine, 2.0 g; glycine, 2.4 g; L-histidine, 2.4 g; L-isoleucine, 6 g; L-leucine, 10.1 g; L-lysine, 6.7 g; L-methionine, 2.3 g; L-proline, 9.2 g; L-phenylalanine, 4.3 g; L-serine, 5.2 g; L-threonine, 4.9 g; L-tyrosine, 5.2 g; and L-valine, 6.7 g. Total: 75.2 g. Placebo: Same as above, plus 3.0 g of L-tryptophan. Total: 78.2 g. For females, 20% reductions in the above quantities were used to account for lower body weight. The drinks were prepared by stirring the mixture and lemon-lime flavoring into 200-ml tap water.

Analysis of plasma samples

Blood samples (10 ml) were analyzed to determine the total plasma tryptophan level and the ratio of tryptophan to other large neutral amino acids (TRP:ΣLNAA ratio). Specifically, the ratio was calculated from the serum concentrations of total tryptophan divided by the sum of the

large neutral amino acids (tyrosine, phenylalanine, valine, isoleucine, leucine). This is important because the uptake of tryptophan in the brain is strongly associated with the amounts of other competing LNAAs due to non-specific transport across the blood-brain barrier. Venous samples were taken in lithium heparin tubes and stored at −20°C. Plasma tryptophan concentrations were determined by an isocratic high-performance liquid chromatography (HPLC) method of analysis. Plasma proteins were removed by precipitation with 3% trichloroacetic acid and centrifugation at 3,000 rpm, 4°C degrees for 10 min, then pipetted into heparin aliquots. An aliquot was diluted in mobile phase before injection onto the HPLC analytical column. Fluorescence end-point detection was used to identify tryptophan.

Supplemental Results

Self-report mood

Consistent with previous studies in healthy volunteers, ATD did not affect subjects' self-reported mood (4-6). PANAS scores were analyzed immediately before drink ingestion and immediately before fMRI scanning. A repeated-measures analysis of variance (ANOVA) with treatment (tryptophan depletion, placebo) and time point (baseline, +5.5 h) as within-subjects factors found no significant effects of treatment, time point, or their interaction on PANAS positive affect (all $p > 0.13$) or negative affect (all $p > 0.15$).

Serotonin manipulation

Additional simple effects analyses showed a significant decrease in plasma tryptophan levels ($t_{(18)} = 13.8$, $p < 0.0001$) on the tryptophan depletion session, averaging 66%. There was also a significant decrease in TRP:ΣLNAA ratio ($t_{(18)} = 12.4$, $p < 0.001$) on the tryptophan depletion session, averaging 85%. In the placebo session, plasma tryptophan levels increased by an average of 88% ($t_{(18)} = -6.2$, $p < 0.0001$); there was no significant change in TRP:ΣLNAA ratios ($t_{(18)} = 0.5$, $p = 0.598$).

fMRI task behavioral results and emotional ratings of facial expressions

Accuracy or correct reaction times on the gender discrimination task were submitted to a 2 x 3 ANOVA examining treatment and expression. Neither measure showed an effect of treatment (accuracy, $F_{(1,18)} = 2.3$, $p = 0.14$; RT, $F_{(1,18)} = 0.4$, $p = 0.50$) nor treatment by expression interaction (accuracy, $F_{(1,18)} = 1.4$, $p = 0.27$; RT, $F_{(1,18)} = 1.2$, $p = 0.31$) (Figure S5A, S5B). Emotional ratings of facial expressions obtained after each scanning session were submitted to a 2 x 3 ANOVA examining treatment and expression which showed no main effect of treatment (ratings for anger, $F_{(1,18)} = 1.3$, $p = 0.26$; ratings for sadness, $F_{(1,18)} = 1.8$, $p = 0.19$) nor treatment by expression interaction (ratings for anger, $F_{(1,18)} = 1.3$, $p = 0.30$; ratings for sadness, $F_{(1,18)} = 2.3$, $p = 0.12$) (Figures S5C, S5D).

Psycho-physiological-interaction (PPI) general linear model (GLM) results

Here, we report: 1) the PPI results obtained when using the left amygdala seed for the contrast angry vs. neutral faces (Montreal Neurological Institute (MNI) local maxima for anterior cingulate cortex (ACC): x 14, y 30, z 18; $t = 3.9$; $p = 0.054$, family-wise error (FWE), small volume correction (svc); ventro-lateral prefrontal cortex (VLPFC): x 54, y 40, z 4; $t = 4.0$; $p = 0.06$, FWE, svc); 2) the PPI results for the angry vs. sad contrast for both the right and left amygdala seed (right amygdala seed, MNI local maxima ventral ACC (vACC): x 10, y 40, z -2; $t = 4.1$; $p < 0.05$, FWE, svc; orbitofrontal cortex (OFC): x 30, y 34, z 18; $t = 4.1$; $p < 0.04$, FWE, svc; left amygdala seed, MNI local maxima vACC: x 10, y 42, z 4; $t = 4.0$; $p = 0.05$, FWE, svc; VLPFC: x 54, y 36, z 2; $t = 4.1$; $p = 0.06$, FWE, svc; OFC: x 30, y 36, z 16; $t = 4.3$; $p < 0.04$, FWE, svc); 3) the PPI GLM during placebo alone (negative change in connectivity) (vACC: x 10, y 40, z 0; $t = 2.58$; $p < 0.005$, uncorrected; VLPFC: x 48, y 26, z 12; $t = 4.79$; $p < 0.05$, FWE, svc) (one sample *t*-test); 4) the PPI GLM during ATD alone (positive change in connectivity) (vACC: x 12, y 48, z -2; $t = 4.55$; $p < 0.05$, FWE, svc; VLPFC: x 36, y 44, z 2; $t = 2.88$; $p = 0.005$, uncorrected) (one sample *t*-test); 5) the PPI for the interaction between treatment (ATD,

placebo) and sex and for the interaction between treatment and tryptophan plasma levels (no significant regions were detected within the region of interest (ROI) at $p < 0.05$ FWE, svc or on other regions outside the ROI at $p < 0.001$, uncorrected, > 10 contiguous voxels); 6) the PPI correlation with individual differences in Behavioral Approach System-drive (for the right and left amygdala seed) under placebo and ATD, separately (Figures S6 and S7).

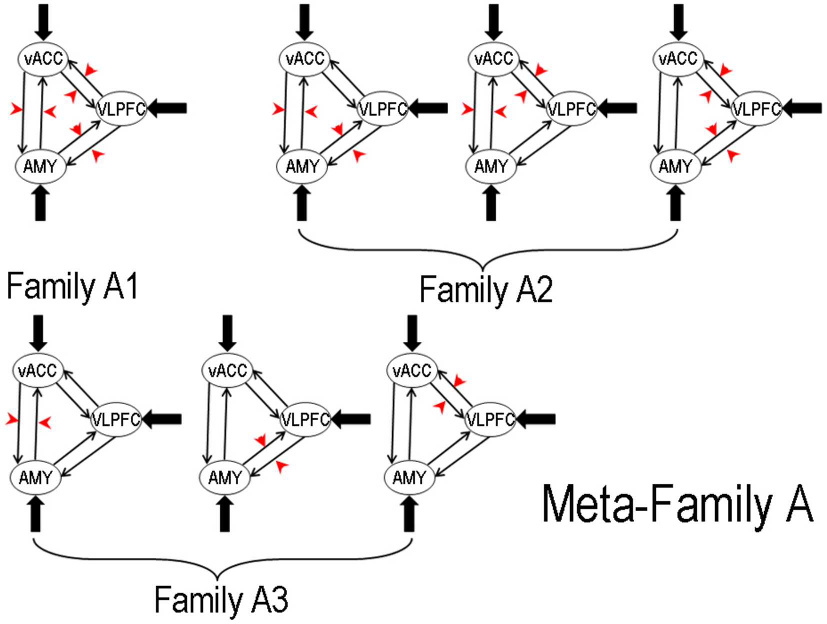

Figure S1. Meta-Family A gathers families where driving inputs (all faces > fixation, black 'thick' arrows) enter the network via parallel inputs into ventral anterior cingulate cortex (vACC), ventro-lateral prefrontal cortex (VLPFC) and amygdala (AMY), simultaneously. Intrinsic connectivity (black 'thin' arrows) represents coupling between regions irrespective of any experimental manipulation. The number and locations of contextual modulators (red arrows, angry > neutral faces) that influence specific neural pathways further define families A1, A2 and A3.

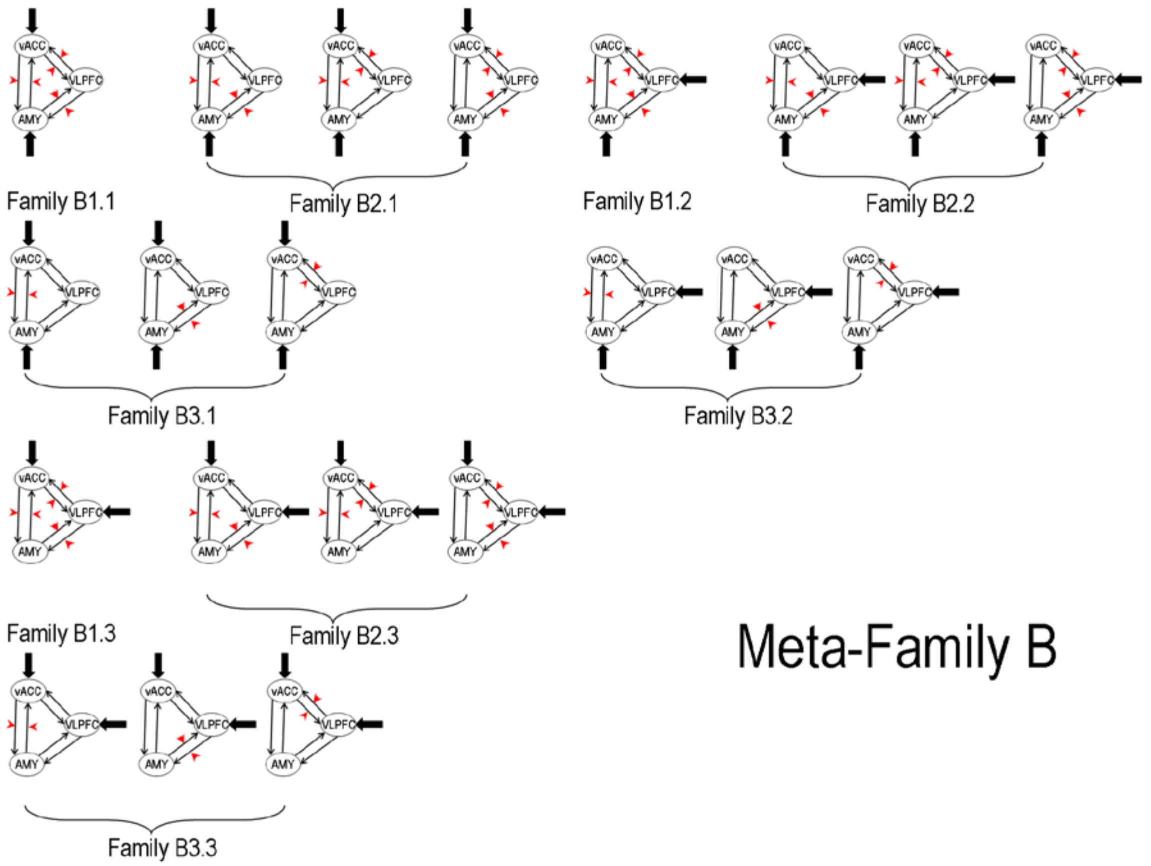

Figure S2. Meta-Family B incorporates families where driving inputs (all faces > fixation, black 'thick' arrows) enter the network via 2 regions (either the ventral anterior cingulate cortex (vACC) and the amygdala (AMY) or the ventro-lateral prefrontal cortex (VLPFC) and AMY or the vACC and the VLPFC). Families are further characterized by the number and locations of contextual modulators (red arrows, angry > neutral) that influence specific neural pathways. This leads to a total of 21 families. Intrinsic connectivity (black 'thin' arrows) represents the coupling between regions irrespective of any experimental manipulation.

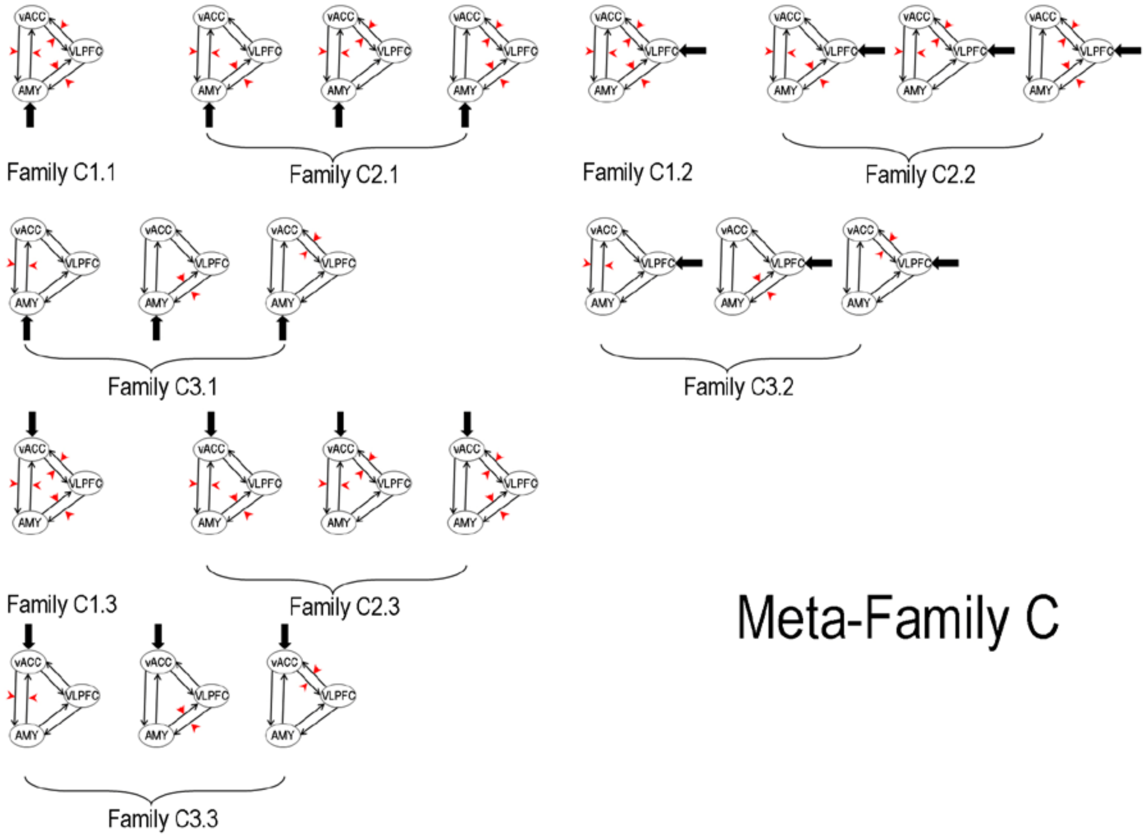

Figure S3. Meta-Family C includes families where driving inputs (all faces > fixation, black 'thick' arrows) enter the network via only 1 region (either the amygdala (AMY) or the ventro-lateral prefrontal cortex (VLPFC) or the ventral anterior cingulate cortex (vACC)). Families are further divided on the basis of the number and locations of contextual modulator (red arrows, angry > neutral) that influence specific neural pathways. This leads to a total of 21 families. Intrinsic connectivity (black 'thin' arrows) represents the coupling between regions irrespective of any experimental manipulation.

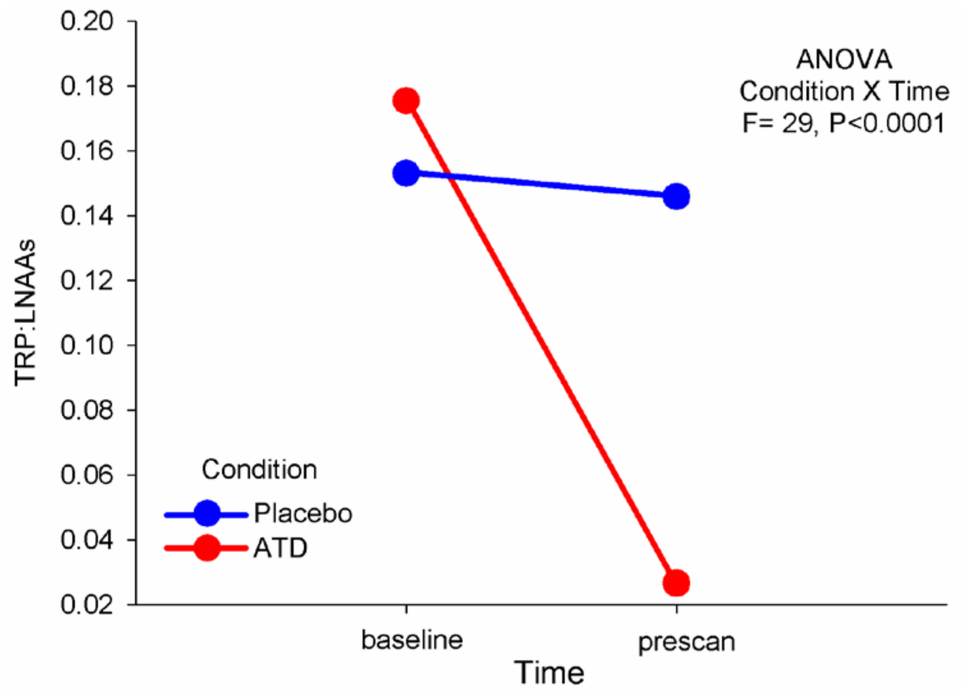

Figure S4. At 5.5 hours (prescan) after acute tryptophan depletion (ATD) there was a significant reduction, relatively to placebo, of the ratio between tryptophan and other large neutral amino acids (TRP:LNAA). See supplementary results for statistics. ANOVA, analysis of variance.

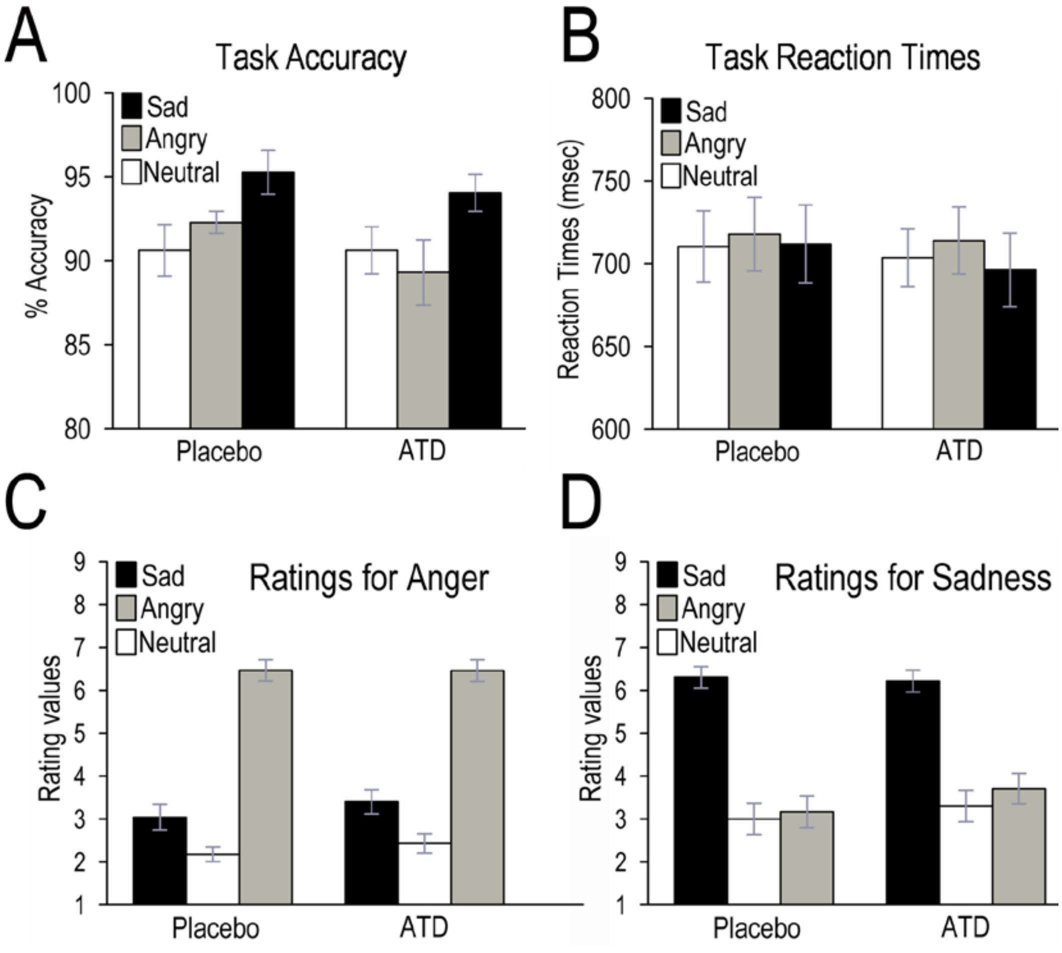

Figure S5. (A, B) Plots of percentage accuracy and reaction times when categorizing the gender of angry, sad and neutral faces during the functional neuroimaging (fMRI) task. See supplemental results for statistics. **(C, D)** Plots of the ratings, obtained outside the scanner, for anger and sadness of all facial expressions used during the fMRI task. See supplemental results for statistics. ATD, acute tryptophan depletion.

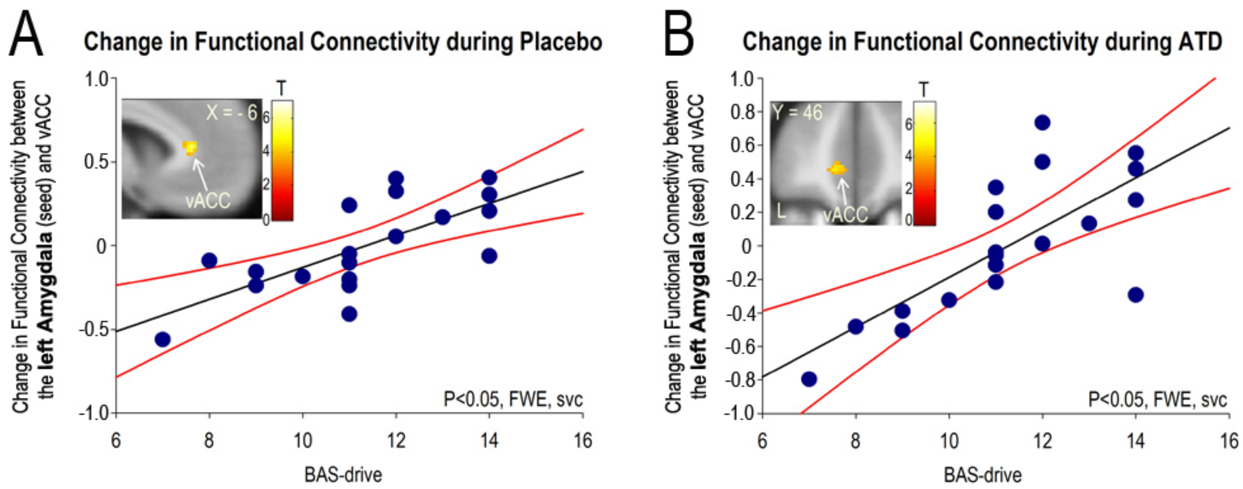

Figure S6. (A) Data plot for the PPI shown in the inset figure under placebo. There is a statistically significant positive correlation (p < 0.05 FWE, svc) between the PPI (i.e. the left amygdala-ventral ACC connectivity as function of the angry versus neutral context) and individual differences in reward–drive score. **(B)** Data plot for the PPI shown in the inset figure under ATD. As for the placebo condition, there is a statistically significant positive correlation (p < 0.05 FWE, svc) between the PPI (i.e. the left amygdala-ventral ACC connectivity as function of the angry versus neutral context) and individual differences in reward–drive score. The regression line (black) and the 95% confidence intervals (red lines) are shown. ACC, anterior cingulate cortex; ATD, acute tryptophan depletion; BAS–drive: Behavioral Approach System–reward drive subscale; FWE, family-wise error; PPI, psycho-physiological-interaction; svc, small volume correction.

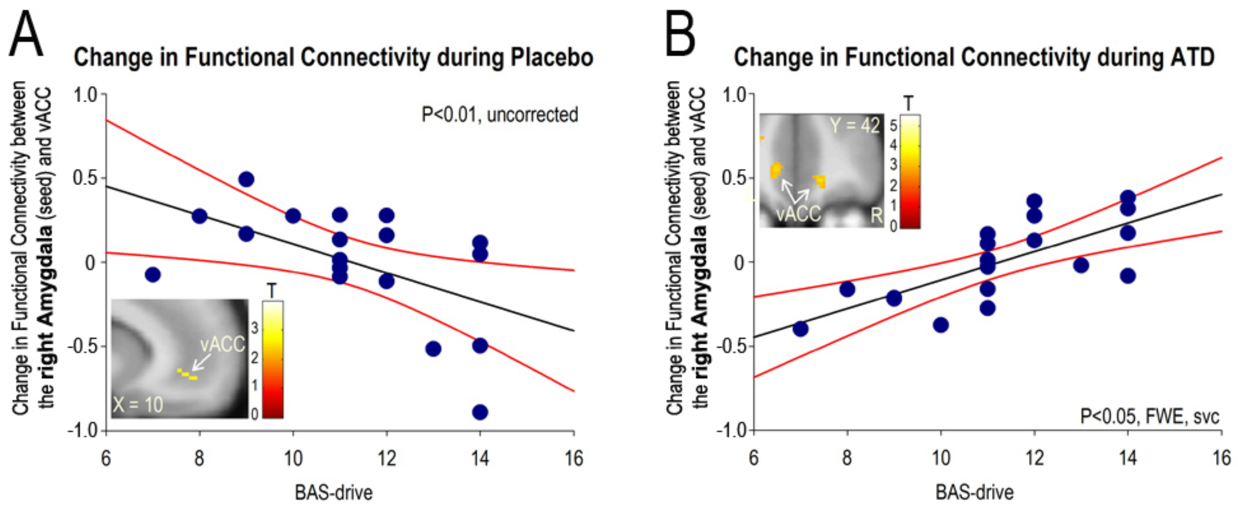

Figure S7. (A) Data plot for the PPI shown in the inset figure under placebo. There is a negative correlation (not meeting our a priori threshold of $p < 0.05$ FWE, svc) between the PPI (i.e. the right amygdala-ventral ACC connectivity as function of the angry versus neutral context) and individual differences in reward–drive score. **(B)** Data plot for the PPI shown in the inset figure under ATD. In contrast to the placebo condition, there is a statistically significant positive correlation ($p < 0.05$ FWE, svc) between the PPI (i.e. the right amygdala-ventral ACC connectivity as function of the angry versus neutral context) and individual differences in reward–drive score. The regression line (black) and the 95% confidence intervals (red lines) are shown. See Figure S6 for abbreviations.

Table S1. Main effect of the task for the contrast angry vs. neutral faces during placebo and acute tryptophan depletion, independently (one-sample *t*-tests).

Cerebral Regions	Hemisphere	T local maxima	MNI coordinates		
			X	Y	Z
Placebo					
VLPFC/Anterior Insula	R	4.56[a]	52	32	4
VLPFC/Anterior Insula	L	3.37[a]	-42	20	0
Amygdala	R	3.76[a]	18	-5	-16
Amygdala	L	3.55[a]	-24	0	-16
Posterior STS	R	3.64	56	-66	4
Posterior STS	L	3.27	-40	-60	8
Extra-striate Cortex	R	3.54	34	-90	2
Extra-striate Cortex	L	3.12	-34	-88	10
Acute Tryptophan Depletion					
VLPFC	R	5.97[a]	46	32	-6
VLPFC	L	4.90[a]	-34	32	-6
Amygdala	R	4.12[a]	30	-8	-22
Amygdala	L	4.02[a]	-22	2	-26
Posterior STS	R	5.15	40	-46	8
Posterior STS	L	4.55	-50	-60	8
Extra-striate Cortex	L	4.18	-36	-82	0

L, left; MNI, Montreal Neurological Institute; R, right; STS, superior temporal sulcus; VLPFC, ventro-lateral prefrontal cortex.

[a]$p < 0.05$, family wise error (small volume correction). Other regions met the criteria of $p < 0.001$, uncorrected, for > 10 contiguous voxels.

Table S2. Main effect of the task for the contrast sad vs. neutral faces during placebo and acute tryptophan depletion, independently (one-sample *t*-test).

Cerebral Regions	Hemisphere	T local maxima	MNI coordinates		
			X	Y	Z
Placebo					
VLPFC/Anterior Insula	L	3.10[a]	-54	20	-6
OFC	L	5.83[a]	-22	44	-14
Posterior STS	R	3.18	-60	-56	6
Posterior STS	L	3.32	54	-46	6
Extra-striate Cortex	R	3.76	24	-98	24
Acute Tryptophan Depletion					
VLPFC	L	4.03[a]	54	32	-2
OFC	L	3.76[a]	2	38	-22
Posterior STS	R	4.41	50	-42	4
Posterior STS	L	3.17	-50	-46	4

L, left; MNI, Montreal Neurological Institute; OFC, orbitofrontal cortex; R, right; STS, superior temporal sulcus; VLPFC, ventro-lateral prefrontal cortex.

[a]$p < 0.05$, family wise error (small volume correction). Other regions met the criteria of $p < 0.001$, uncorrected, for > 10 contiguous voxels.

Table S3. Main effect of the task for the contrast angry vs. sad faces during placebo and acute tryptophan depletion, independently (one-sample t-test).

Cerebral Regions	Hemisphere	T local maxima	MNI coordinates		
			X	Y	Z
Placebo					
VLPFC	R	4.94[a]	42	34	-8
VLPFC/Anterior Insula	L	5.53[a]	-28	30	-8
Amygdala	R	4.57[a]	18	-5	-16
Amygdala	L	4.70[a]	-20	-4	-16
Acute Tryptophan Depletion					
VLPFC	R	3.94[a]	40	38	-2
VLPFC/Anterior Insula	L	4.37[a]	-30	20	2
Amygdala	R	3.14[a]	28	0	-28
Amygdala	L	2.99[a]	-26	0	-24

L, left; MNI, Montreal Neurological Institute; R, right; VLPFC, ventro-lateral prefrontal cortex.
[a]$p < 0.05$, family wise error (small volume correction).

Supplemental References

1. Watson JB (1988): A comparison of stutterers' and nonstutterers' affective, cognitive, and behavioral self-reports. *J Speech Hear Res*. 31:377-385.

2. Carpenter LL, Anderson GM, Pelton GH, Gudin JA, Kirwin PD, Price LH, *et al.* (1998): Tryptophan depletion during continuous CSF sampling in healthy human subjects. *Neuropsychopharmacology*. 19:26-35.

3. Young SN, Smith SE, Pihl RO, Ervin FR (1985): Tryptophan depletion causes a rapid lowering of mood in normal males. *Psychopharmacology (Berl)*. 87:173-177.

4. Cools R, Calder AJ, Lawrence AD, Clark L, Bullmore E, Robbins TW (2005): Individual differences in threat sensitivity predict serotonergic modulation of amygdala response to fearful faces. *Psychopharmacology (Berl)*. 180:670-679.

5. Cools R, Robinson OJ, Sahakian B (2008): Acute tryptophan depletion in healthy volunteers enhances punishment prediction but does not affect reward prediction. *Neuropsychopharmacology*. 33:2291-2299.

6. Evers EA, Tillie DE, van der Veen FM, Lieben CK, Jolles J, Deutz NE, *et al.* (2005): Effects of a novel method of acute tryptophan depletion on plasma tryptophan and cognitive performance in healthy volunteers. *Psychopharmacology (Berl)*. 178:92-99.
